# Mapping the path to diversity in clinical trials: a qualitative study of community member and stakeholder perspectives

**DOI:** 10.1186/s12889-025-25574-z

**Published:** 2025-12-09

**Authors:** Meera J. Patel, Ketan Tamirisa, Emilie Ruiz, Zahria Griggs, Eunice Huan, Heather Kitzman

**Affiliations:** 1 Peter J. O’Donnell Jr. School of Public Health, UT Southwestern Medical Center, Dallas, TX 75390 USA; 2 Washington University in St. Louis, St. Louis, MO 63130 USA

**Keywords:** Clinical trials, Participant diversity, UREG, Community members, Research stakeholders

## Abstract

**Background:**

Patient diversity in clinical trials has been a critical issue in the advancement of translational scientific research. There is a dire need for better representation of all populations to understand the efficacy of novel therapeutics and health interventions for promising outcomes. The current study aims to identify key factors that may lead to equitable participation in clinical research.

**Methods:**

This qualitative study included focus groups with lower-income, racially or ethnically diverse community members (*n* = 51) and research stakeholders (*n* = 50) to identify factors that influence clinical trial participation. Focus groups were conducted between November 2023 and May 2024 within community-settings in Dallas, Texas. Directed Content Analysis using the Socioecological Model (SEM) as the theoretical framework was utilized for data analysis.

**Results:**

Key barriers, facilitators, and implementation strategies to clinical trial participation across four levels – individual, interpersonal, community, and macroeconomic/policy – were identified from the perspectives of community members and research stakeholders, separately. Prominent barriers included fear and mistrust at the individual level; misconceptions at the interpersonal level; historical experiences (defined as collective mistreatment faced by the participant’s community), lack of knowledge, and logistics at the community level; systemic mistrust as well as racism and consent/study language at the macroeconomic/policy level. Facilitators included personal health knowledge, monetary incentives, and safety at the individual level; trusted relationships and family health knowledge at the interpersonal level; altruism at the community level; transparency of safety/risk at the macroeconomic/policy level. Implementation strategies proposed were rebuilding trust, building relationships through community engagement, increasing awareness about clinical research, and leveraging culturally tailored resources to promote inclusiveness.

**Conclusions:**

Underrepresentation of diverse populations in clinical studies warrants the need to consider multifaceted approaches to mitigate the current public health crisis. Ultimately, building trust, community engagement to promote knowledge and awareness, and transparency are essential for improving diversity in clinical trials.

**Supplementary Information:**

The online version contains supplementary material available at 10.1186/s12889-025-25574-z.

## Background

Clinical trials are crucial for evaluating the efficacy and safety of therapeutic options, improving disease prevention methods, informing guidelines and standards of care, and promoting better health and quality of life. Outcomes from these trials can vary among specific patient populations, including those defined by race and ethnicity or socioeconomic status [1, 2]. However, a lack of diverse representation persists in contemporary clinical trials, ultimately leading to limitations in scientific knowledge and advancement. Between 2015 and 2019, out of 688 randomized clinical trials published in high-tiered journals, such as *Journal of American Medical Association*, *The Lancet*, and the *New England Journal of Medicine*, only 50% reported race and ethnicity characteristics [3]. Of those, enrollment was high among White participants (median [IQR]: 77–84% [67%−91%]) as compared to the approximate 5% enrollment of Black and Hispanic persons [3].

Regrettably, this disparity remains consistent across numerous medical and pharmaceutical studies, which poses a significant limitation in the improvement of health outcomes and health equity for all individuals. For example, among the 50,411 patients enrolled in oncology trials evaluating the efficacy of treatment modalities between 2017 and 2022, only 4.4% participants were Black persons and 4.2% were individuals of Hispanic/Latinx origins [4]. Indigenous populations are severely underrepresented in clinical trials, where across 1,800 clinical studies, American Indian/Alaska Natives have remained at a disproportionately low level of representation – 1% of all participating individuals [5]. In stroke-focused clinical trials, Black (22.7%) and Hispanic (5%) individuals were strikingly underrepresented, where low enrollment was discordant to the actual burden of stroke in these groups [6]. Another study, which included 24 new treatment drugs for cardiovascular disease approved by the FDA between 2006 and 2020, enrolled a total of 187,294 participants of which only 3% were Black persons [7]. Unfortunately, most of these clinical trials are relevant to racial and ethnic minorities who are predisposed to higher risks for such diseases, yet the limitation of a diverse participant pool in research hinders the ability to identify and understand the biological mechanistic variations that may impact health outcomes [8].

Disparities in research participation stem from a wide range of barriers that potentially hinder underrepresented racial and ethnic group (UREG) participants’ willingness and ability to partake in clinical trials. Examples of these obstacles include basic logistics, such as transportation, lodging, childcare, and meals [9], which can transform into motivators if overcome. In recent years, efforts to improve representation of UREG in clinical trials have been initiated [10]. In collaboration with patients and researchers, the American Association for Cancer Research created a biennial report for establishing equitable enrollment in cancer [11], whereas the FDA designed a guidance protocol to increase UREG and female representation in clinical research [12]. Furthermore, Research Match, a non-profit program funded by the National Institute of Health (NIH), was implemented to directly connect patient volunteers with clinical trials via research collaborators, including healthcare institutions [13]. Despite these advancements in clinical trial enrollment and research, the rate of participation has not changed substantially over time [14]. Therefore, to increase accessibility and interest in clinical trial research, it is important to consider the intersection of race and ethnicity with social determinants of health, such as transportation, health literacy, healthcare access, and employment restrictions.

Clark et al. investigated stakeholder feedback to develop communication strategies to improve UREG representation in clinical trials [15]. Building on their findings, our qualitative study identified key factors that hinder and promote diversity in clinical trials using perspectives of both community members and research stakeholders. Understanding the disparity of UREG representation in clinical research is a multifaceted challenge and requires a comprehensive social and behavioral lens to identify and address the lack of participation. Engagement and participation in research have many layers, such as motivation within a person, influence of close relationships, impact of the community one identifies with, and systemic policies set in place. Therefore, adapting Bronfenbrenner’s Socioecological Model (SEM) as a theoretical framework [16], we recognized the prominent barriers, facilitators, and implementation strategies impacting UREG. The SEM emphasizes that one’s behavior and health outcomes are shaped by a complex interplay of multiple levels - from individual to interpersonal, community, and macroeconomic/policy [17]. McLeroy et al. highlight how changes in one’s social environment, which includes interpersonal relationships, organization, community, and system policies, can influence changes in an individual’s behavior as well as health outcomes [17]. In this manuscript, we explored these layers of connections among UREG participants to better understand areas of improvement, strengthening, and strategies for clinical research participation.

## Methods

### Recruitment

This study was approved by the University of Texas Southwestern Medical Center (UTSW) Institutional Review Board. The community member focus group (FG) participants were low-income residents of Dallas, Texas and primarily from urban communities. Recruitment of community members for FGs included public announcements and dissemination of study flyers by partnering with community organizations, such as local churches, recreation centers, and housing complexes, that we have long-standing partnerships with through a North Texas Community Health Coalition (NTCHC), and which serve predominantly urban communities experiencing poverty that are medically underserved in Dallas, Texas. Interested individuals were screened for eligibility by telephone using the following inclusion criteria: 1) ≥ 18 years of age, 2) self-identified as from a UREG, 3) no prior participation in clinical trials, and 4) spoke English or Spanish.

For the research stakeholder focus groups (FGs), community organization leaders and research staff from UTSW were recruited through existing relationships. Community stakeholder recruitment targeted those from NTCHC organizations that provide services to populations experiencing poverty, including faith-based entities embedded within urban areas of Dallas. Community stakeholders were identified as trusted leaders within their communities and invited to participate through direct outreach by the study team or through community partner referrals. Another group of stakeholders included UTSW research staff. UTSW, a large academic medical center that conducts numerous clinical trials across diverse medical specialties, employs research staff with extensive expertise in study implementation, including research operations, recruitment strategies, and community engagement. UTSW stakeholders, including an IRB regulatory specialist, clinical trial navigators, project coordinators, and research assistants, were invited based on their experience and knowledge of participant recruitment and engagement in clinical research with UREG populations. To be eligible, individuals had to represent one of these stakeholder groups, be ≥ 18 years of age, and speak English or Spanish.

Nine FGs, four with community members and five with research stakeholders, were conducted in community-based settings to foster an environment where participants felt comfortable engaging in open and honest dialogue.

### Adaptation of the socioecological model (SEM)

We applied Bronfenbrenner’s SEM [16], which illustrates how individuals are influenced by their social environments and interactions at the individual, interpersonal, community, and macroeconomic/policy level, as the theoretical framework for this study. Questions in the FG guides were informed by findings from Clark et al. [15] and derived from prior qualitative studies that explored barriers, facilitators, and strategies for engaging UREG populations in clinical research [18–24]. Specifically, our FG guides were modified to incorporate probes exploring various aspects of the SEM (Supplementary File). Community and stakeholder FG guides were designed to be complementary in both structure and context, however, questions for the stakeholders were specifically tailored to elicit insights based on experiences with research participants and perspectives on research practices. Overall, the FG guides were framed to understand three main topics: (a) barriers to clinical trial participation, (b) facilitators that motivate clinical trial enrollment, and (c) implementation strategies to increase participant diversity in clinical trials.

### Focus group training

Focus groups were facilitated by research staff, including bilingual community health workers (CHWs), research project coordinators, and graduate students. The facilitators were thoroughly trained by senior researchers experienced in qualitative methods. Research staff training was guided by established qualitative research frameworks and best practices for focus group methodology, particularly Designing and Conducting Focus Group Interviews [25] and Qualitative Methods in Public Health: A Field Guide for Applied Research [26]. The objectives of the training were to understand and implement principles of qualitative data collection, which include use of open-ended questions to develop the FG guide, create an inclusive and comfortable environment, manage group dynamics, minimize moderator bias, and encourage balanced participation.

### Data collection

Each facilitator adhered to the study protocol and utilized the FG guides to ensure consistency in conducting each group session. Community member and stakeholder FGs were conducted separately, each lasting approximately one hour. Sessions were audio recorded, then professionally transcribed by an external agency. Focus groups conducted in Spanish were first transcribed, then translated in English, and lastly validated for accuracy by two bilingual CHWs on the research team. All transcripts were also validated by cross-referencing with the original audio and study team notes for accuracy. Additionally, de-identifiable sociodemographic and socioeconomic information was collected with a self-report survey (Supplementary File).

### Data analysis

Using the principles of the SEM, transcribed qualitative data was coded by hand and evaluated using a deductive-inductive coding approach. Barriers and facilitators described in previous literature [18–24] were considered in developing the initial codebook, which was further refined after reviewing transcripts for accurate data alignment before analysis. Coded data was analyzed via Directed Content Analysis by three racially diverse research staff for inclusive perspectives to mitigate researcher bias via triangulation. The consensus of coded transcripts was at 100% agreement prior to a detailed frequency analysis. Each component was carefully analyzed through the SEM framework to identify factors across four socially-influenced interactive stages: (1) individual level – personal beliefs, attitudes, and views; (2) interpersonal level – an individual’s inner network (i.e., friends, family, providers); (3) community level – an individual’s outer network (i.e., church, neighborhood, community groups); and (4) macroeconomic/policy (macro/policy) level – organizations, institutions, and systemic policies.

## Results

### Characteristics of the focus group participants

Sociodemographic and socioeconomic data of all FG participants (*N* = 101; community members = 51, research stakeholders = 50), including age, sex, race, ethnicity, education, total annual household income, and household size, is presented in Table 1. The mean [standard deviation (SD)] age of the participants was 54.0 [16.0] years, 81.2% were females, 39.6% self-identified as Black/African American persons, and 51.5% self-identified as having Hispanic, Latino, or Spanish origin. Approximately 57% of the participants did not have a college degree, 59% had an annual household income of ≤$50,000, and 28% had a household size of ≥ 5 individuals.

**Table 1:** Socioeconomic characteristics of focus group participants

Variable	All	Community	Stakeholder
N	101	51	50
Age, mean (SD)	54.0 (16.0)	59.9 (15.1)	50.0 (15.6)
Sex, n (%)			
Male	19 (18.8)	4 (7.8)	15 (30.0)
Female	82 (81.2)	47 (92.2)	35 (70.0)
Race/ethnicity, n (%)			
Hispanic/Latino/Spanish Origin	52 (51.5)	21 (41.2)	31 (62.0)
Black/African-American	40 (39.6)	23 (45.1)	17 (34.0)
*Other (*American Indian/Alaska Native, Chinese, Asian Indian, Vietnamese)	10 (9.9)	3 (5.8)	7 (14.0)
Education, n (%)			
Some high school, no diploma	18 (17.8)	16 (31.5)	2 (4.0)
High school diploma	12 (11.9)	6 (11.8)	6 (12.0)
GED or equivalent	3 (3.0)	1 (2.0)	2 (4.0)
Some college	24 (23.8)	14 (27.5)	10 (20.0)
Associate’s degree	11 (10.9)	4 (7.8)	7 (14.0)
Bachelor's degree	17 (16.8)	7 (13.7)	10 (20.0)
Master's degree	14 (13.9)	2 (3.9)	12 (24.0)
Professional School degree	0 (0.0)	0 (0.0)	0 (0.0)
Doctoral degree	2 (2.0)	1 (2.0)	1 (2.0)
Household Income, n (%)			
<$20,000	26 (25.7)	21 (41.2)	5 (10.0)
$20,000 - $34,999	18 (17.8)	10 (19.6)	8 (16.0)
$35,000-$49,999	16 (15.8)	9 (17.6)	7 (14.0)
$50,000-$64,999	8 (7.9)	4 (7.8)	4 (8.0)
$65,000-$79,999	16 (15.8)	4 (7.8)	12 (24.0)
$80,000-$94,999	6 (5.9)	1 (2.0)	5 (10.0)
$95,000 or more	11 (10.9)	2 (3.9)	9 (18.0)
Household Size, n (%)			
1	27 (26.7)	19 (37.3)	8 (16.0)
2	22 (21.8)	12 (23.5)	10 (20.0)
3	12 (11.9)	4 (7.8)	8 (16.0)
4	12 (11.9)	4 (7.8)	8 (16.0)
More than 5	28 (27.7)	12 (23.5)	16 (32.0)

### Barriers to participation in clinical research

Several significant barriers were identified across individual, interpersonal, community, and macro/policy levels by both community members (C) and stakeholders (S). At the individual level, fear (*n* = 20 for C and S groups) and mistrust (*n* = 12 and *n* = 16 for C and S groups, respectively) were identified as the most prominent barriers across both groups (Table 2). Community members mentioned fear of “the unknown…it might kill me” or “what’s going to happen to me six months…down the road” and stakeholders stated fear of “what we don’t know”. The major barrier perceived at the interpersonal level was beliefs (*n* = 50 and *n* = 35 for C and S groups, respectively), including myths and misconceptions (Table 3). For example, community members cited myths such as “they [doctor] are going to test me…not going to do anything”. Stakeholders emphasized how cultural beliefs underly misconceptions such as “becoming the guinea pig” and how trying to address these beliefs is “almost like having to break down a brick wall”. However, at the community level, the most significant barrier varied between perspectives. Community members highlighted historical experiences (*n* = 20) as their primary barrier (defined as collective mistreatment faced by a participant’s community), then lack of knowledge (*n* = 15), and mistrust (*n* = 12), whereas stakeholders identified time (*n* = 20) as the top barrier, then language (*n* = 17) and lack of knowledge (*n* = 17), mistrust (*n* = 14), and lastly historical experiences (*n* = 11) (Table 4). Not surprisingly, mistrust was recognized as a barrier across both perspectives at the macro/policy level (Table 5). According to the community FG, mistrust (*n* = 62) was divided among medical mistrust (*n* = 32), then mistrust toward government (*n* = 17), and lastly mistrust toward pharmaceuticals (*n* = 13). Stakeholders perceived mistrust (*n* = 40) slightly different – medical (*n* = 21), then pharmaceutical (*n* = 10), and lastly government (*n* = 9).

**Table 2:** Individual level analysis for community and stakeholder perspectives

**Community Perspective**			
**Topics **	**Subtopics**	**Frequency**	**Illustrative Quotes**
Barriers	1.1a Mistrust	12	*“And if you put them side by side, you have Guinea pig and you have research. How are you going to convince me you’re not using me as a Guinea pig?” (1.1a, 2.1b)* *“How are you going to come asking me for something you haven’t even addressed my everyday day-to-day needs, right? So I know, for example, in low income, we do suffer from resources.” (1.1c)* *“Being scared of what they are going to do. The unknown. I’m not going to try that because it might kill me.” (1.1a, 1.1d, 4.1a)* *“If I take it now and you get your information and you give me my pat on the back, the ‘attaboy’ and a couple of dollars, what’s going to happen to me six months a year down the road because I took your study? Do I trust you like that? Important.” (1.1d, 4.1a)*
	1.1b Lack of knowledge	3	
	1.1c Basic needs		
	1.1c.i Transportation	4	
	1.1c.ii Housing	2	
	1.1c.iii Utilities (household expenses)	1	
	1.1c.iv Food	2	
	1.1c.v Lack of healthcare	3	
	1.1d Fear	20	
	1.1e Language	3	
Facilitators	1.2a Monetary incentives	31	*“...you just got to do something that’s going to be significant enough that they feel like they’re winning, because they know that there’s an underlying message somewhere and that trust is there.” (1.2a) * *“But I also know the benefit of it (research) too because there's a lot that I wouldn't know about my own health if I didn't participate in some of these things. So to answer your question, yes. But, yes, I know why this information is important.” (1.2b, 1.3b)*
	1.2b Personal health knowledge	26	
	1.2c Safety	9	
Implementation	1.3a Advocacy	4	*“You find time to do what you want to do and what you need to do.” (1.3c)* *“We should be more alert, and aware of what’s going on, and we don’t want to participate in it, we shuffle it off to the side.” (1.3c, 3.3e)*
	1.3b Awareness	8	
	1.3c Self-Accountability	15	

Stakeholder Perspective			
Topics	Subtopics	Frequency	Illustrative Quotes
Barriers	1.1a Mistrust	16	*“[Research is] something that takes something from you and don’t give something to you.” (1.1a)* *“Many of us didn’t know those things were going on as far as the health is, because one, we don’t have the money to go to the center all the time. We don’t have the resources.” (1.1c)* *“And then all the expenses that you might require to come to the clinic so that can be gas, parking, meals...” (1.1c.i, 1.1c.iv)* *“Because again, implicit bias or unconscious bias, we gravitate towards what’s familiar to us and we stay away from what’s maybe not very familiar because we fear what we don’t know or are uncomfortable with what we don’t understand.” (1.1d, 2.3c)*
	1.1b Lack of knowledge	3	
	1.1c Basic needs		
	1.1c.i Transportation	9	
	1.1c.ii Housing	0	
	1.1c.iii Utilities (household expenses)	4	
	1.1c.iv Food	2	
	1.1c.v Lack of healthcare	8	
	1.1d Fear	20	
	1.1e Language	7	
Facilitators	1.2a Monetary incentives	28	*“Or if it’s not altruistic, sometimes people just want money.” (1.2a)* *“It’s like why is the research important to me? I know you’re trying to better the world or whatever help all these other people, but how does that affect me? Why should I participate? (1.2b)*
	1.2b Personal health knowledge	11	
	1.2c Safety	18	
Implementation	1.3a Advocacy	0	*“But the information is there for all, we’ve to do just [inaudible] the information.” (1.3c)*
	1.3b Awareness	1	
	1.3c Self-Accountability	5	

**Table 3:** Interpersonal level analysis for community and stakeholder perspectives

**Community Perspective**			
**Topics**	**Subtopics**	**Frequency**	**Illustrative Quotes**
Barriers	2.1a Mistrust (providers)	15	*“In my estimation, we’re the Guinea pigs for white people... .” (2.1b, 3.1a, 4.1b)* *“Hispanic men is very proud. He didn’t want to see a doctor, he didn’t want to give money. He said: ‘They are going to test me and, in the end, they are not going to do anything.’ That’s the problem with Hispanic men.” (2.1a)*
	2.1b Beliefs (myths/misconceptions)	50	
	2.1c Lack of awareness	7	
	2.1d Lack of transparency	13	
Facilitators	2.2a Trusted relationship	21	*“And now I know that, ‘Hey, I literally have peers that are in the medical industry that I can actually talk to and get more information about things that my parents didn’t have access to.” (2.2a, 2.2b, 4.3b.ii)*
	2.2b Knowledge	2	
	2.2c Family health knowledge	10	
Implementation	2.3a Advocacy	3	*“Having people like you, who come talk with the community. That motivates. That’s the most important because we know you are trying to do something good. The research business (inaudible). You have to reach us.” (2.3c, 4.3c.i)*
	2.3b Transparency	4	
	2.3c Identify with individual	10	

**Stakeholder Perspective**			
**Topics**	**Subtopics**	**Frequency**	**Illustrative Quotes**
Barriers	2.1a Mistrust (providers)	6	*“General mistrust of health care professionals.” (2.1a)* *“And so when you try to overcome those miscommunication barriers, it's almost like having to break down a brick wall because it's so embedded culturally speaking that it's really hard, especially with the elders, the older community that they're like don't talk to me about that. I don't want to hear anything about it.” (2.1b, 3.1b)* *“Obviously the need is out there and people are wanting to reach out, but they don’t know how.” (2.1c)*
	2.1b Beliefs (myths/misconceptions)	35	
	2.1c Lack of awareness	14	
	2.1d Lack of transparency	4	
Facilitators	2.2a Trusted relationship	44	*“And then again, they’ve got to be somebody they trust.” (2.2a)* *“I just think it will provide knowledge or information for people that don't know what's going on with medical issues or medications.” (2.2b)*
	2.2b Knowledge	6	
	2.2c Family health knowledge	4	
Implementation	2.3a Advocacy	3	*“I think so, I think people need a degree of trust to be able to agree to join and seeing somebody that looks like you is going to help along” (2.3c)*
	2.3b Transparency	11	
	2.3c Identify with individual	39	

**Table 4:** Community level analysis for community and stakeholder perspectives

**Community Perspective**			
**Topics **	**Subtopics**	**Frequency**	**Illustrative Quotes**
Barriers	3.1a Mistrust	12	*“And that’s because historically, African American people have been misled, misinformed, under-informed, has sustained injury when it comes to clinical trials and studies. We haven’t been put – and this is beyond me.” (3.1b)* *“Well, there is something very important, even though we are not the minority, Hispanics are at a low level, and I think that also depends on the culture we have because we go through traumatic events, for example, being immigrants.” (3.1b, 4.1a.iii)* *“Because we are not very well educated. And we haven't had the education that this generation is starting to have.” (3.1c)*
	3.1b Historical experiences	20	
	3.1c Lack of knowledge	15	
	3.1d Language	2	
	3.1e Time	1	
Facilitators	3.2a Improvement of health		*“But if we participate more, then we’ll know what medicine is best for our particular race.” (3.2a.ii)* *“Me, myself, like diabetes, high blood pressure, sugar diabetes, travel from generation to generation. I would participate in a thing like that if it would help stop these diseases from destroying our people.” (3.2a.ii)*
	3.2a.i Generational health	7	
	3.2a.ii Community health	28	
	3.2b Acknowledgement of history	5	
	3.2c Trusted relationship (faith, belief system)	4	
	3.2d Minimal time	0	
Implementation	3.3a Advocacy	11	*“I think that one of the benefits is to bring awareness to the communities. Because every race suffers from different illness.” (3.2a.ii, 3.3e)* *“So with information and education, I imagine that word ‘research’ wouldn’t be so strong.” (3.3d, 3.3e)*
	3.3b Inclusion	12	
	3.3c Community outreach	18	
	3.3d Educate	30	
	3.3e Awareness	31	
	3.3f Accessibility	4	

**Stakeholder Perspective**			
**Topics **	**Subtopics**	**Frequency**	**Illustrative Quotes**
Barriers	3.1a Mistrust	14	*“I also say, it depends on who's even presenting the information because if somebody don't look like me presenting the information, I'm skeptical about the information because I already know in my mind not too many black folks, 3% participate in clinical trials. That's something we don't do.” (1.1a, 3.1a)* *“But also like the history of that, if the participant knows a little bit about the history of America, they might be hesitant to even think about participating too.” (3.1b)* *“...but if there’s a large time commitment, I don’t know if they’re willing to give that time.” (3.1e)*
	3.1b Historical experiences	11	
	3.1c Lack of knowledge	17	
	3.1d Language	17	
	3.1e Time	20	
Facilitators	3.2a Improvement of health		*“There could be other factors too, maybe if it's a research study that pertains to something that's personal to you, like let's say you had family history of cancer and you have cancer and you see a clinical trial in cancer and you're like, I want to participate to help other cancer patients in the future. [crosstalk] I think that might be another reason to participate.” (2.2c, 3.2a.ii)* *“What I think too is we would use clergymen, they could address their congregation and say, hey, everybody needs to get screened for colon cancer. Why? Because these church members already have a trust and a rapport with their clergyman. So I'm going to trust you because I've been coming to your church for 10 years.” (3.2c)* *“I think studies that have short duration, like one-time survey or one-time interview.” (3.2d)*
	3.2a.i Generational health	2	
	3.2a.ii Community health	21	
	3.2b Acknowledgement of history	1	
	3.2c Trusted relationship (faith, belief system)	6	
	3.2d Minimal time	6	
Implementation	3.3a Advocacy	4	*“But also engagement, making sure that they're engaged, they're informed, they're invited, they're included.” (3.3b, 3.3c, 3.3d)* *“So, bring it to the table, bring it to the community.” (3.3c)* *“...utilize social media.” (3.3c, 3.3e, 4.3b.ii)* *“They ain't going to do nothing. So, like [redacted] said, making them aware that it's available to you, make it in a location like this, I thought it to be very convenient when it's in the community.” (3.3b, 3.3e, 3.3f)*
	3.3b Inclusion	28	
	3.3c Community outreach	50	
	3.3d Educate	32	
	3.3e Awareness	27	
	3.3f Accessibility	33	

**Table 5:** Macroeconomic/policy level analysis for community and stakeholder perspectives

**Community Perspective**			
**Topics **	**Subtopics**	**Frequency**	**Illustrative Quotes**
Barriers	4.1a Mistrust		*“If I get my food stamps taken away, why would I want the government or some medical person to talk to me about my health when I live in a food dessert?” (4.1a.i, 4.1a.iii)* *“I think you come in my neighborhood to get something because that’s the only time you come, when you want something.” (4.1a.i, 4.1a.iii)* *“He went to the (inaudible) hospital. When we get there, they told him ‘go home and die, we can’t do anything’. And they explored him, they opened him up, and then they shut him down.” (4.1a.i)*
	4.1a.i Medical	32	
	4.1a.ii Pharmaceutical	13	
	4.1a.iii Government	17	
	4.1b Racism	14	
	4.1c Knowledge	4	
	4.1d Health policy (quality of care)	6	
	4.1e Consent/study language	1	
	4.1f Citizenship	1	
Facilitators	4.2a Knowledge		*“It would call my attention, to participate, not being paid. If I’m receiving a treatment, that would call my attention.” (4.2a.i)* *“But you’ve got to be convinced of what you’re talking about and assure me that it’s all safe and it’s all good.” (1.2c, 4.2b.iii)* *“But I think first and foremost, we have to acknowledge why the African American community is reluctant to participate in these studies and respond to that reluctance.” (3.1a, 4.2c)* *“If they tell me that I can be part of this, and I’m going to receive treatment for an illness, and I don’t have to pay for it, I would be like...because is so expensive.” (4.2d)*
	4.2a.i Evidence-based findings	6	
	4.2b Transparency		
	4.2b.i Clear motive	5	
	4.2b.ii Open communication	6	
	4.2b.iii Safety and risk (security)	10	
	4.2c Acknowledgement of history	7	
	4.2d Pharmaceutical coverage	5	
Implementation	4.3a Advocacy	0	*“Something like to that effect, the advertisement. Or people going out to the community centers, like (inaudible) reach to us.” (4.3c.ii)* *“I would have to make sure I got these flyers, and everything, where you got to go, what time it’s going to be, and find out if they can get there in any way if I can do it, I’ll make sure they get there.” (4.3b.ii)* *“A lot of research is done, here in the USA, in one language. Is sad to say it, but the translation is not always exact to what people can understand when is translated to the native tongue.” (3.1d, 4.3d.i)* *“Finding a different word because we already have a negative association with research and clinical trials.” (4.3e)*
	4.3b Accessibility		
	4.3b.i Remote health	3	
	4.3b.ii Access to information	12	
	4.3c Community outreach		
	4.3c.i Hire diverse CHWs	1	
	4.3c.ii Build relationships with community (equal partnerships)	11	
	4.3c.iii Comprehendible educational material	3	
	4.3d Inclusion		
	4.3d.i Culturally translated material	2	
	4.3e Terminology revision	5	

**Stakeholder Perspective**			
**Topics **	**Subtopics**	**Frequency**	**Illustrative Quotes**
Barriers	4.1a Mistrust		*“Because if you are on the phone or see an email: ‘We are UT Southwestern’, I feel like, oh you guys are just researcher in your ivory tower.” (4.1a.i)* *“I wouldn’t participate in anything that I have to take medication.” (1.1d, 4.1a.ii)* *“People are very reluctant. Even before 9/11, when they start asking you, where are you going, and what's your Social Security number, and where are your mom and dad from? And I'm like, I don't even know my dad.” (4.1a.iii)* *“Okay, because I never know there's a lot of politics and things, it could be the government putting money to you all to do this to us.” (4.1a.iii)* *“I wonder if also another barrier would be especially for Latinos, or even our immigrants from Somalia or Nigeria, that immigration status, they don't want to fool with anything, giving information or being a part of something, because they might check their immigration status.” (4.1f)*
	4.1a.i Medical	21	
	4.1a.ii Pharmaceutical	10	
	4.1a.iii Government	9	
	4.1b Racism	1	
	4.1c Knowledge	6	
	4.1d Health policy (quality of care)	14	
	4.1e Consent/study language	15	
	4.1f Citizenship	5	
Facilitators	4.2a Knowledge		*“I also think it’s important to tell them what it is, but also provide examples of positives that come from it.” (4.2a.i)* *“And proof. Let me see the proof. That’s the fact that this is not what I heard.” (4.2a.i)* *“Yes, transparency. Having clear information.” (4.2b.ii)* *“Before we finish, I know a lot of people go to the libraries and ask questions. And for us, it's very important to know when you talk about transparency to have that information, for example, people will come and say, like he was saying, if I'm an immigrant, if I have insurance or not, can I participate in the trial? So, those questions are important to let people know, hey, you need this, or you don't need it.” (4.2b.ii)* *“So when I say reprogramming as far as re-educating, it's not so much as there's a lack of mistrust, and by right, I can understand why it is a mistrust. I'm going to be honest, even myself certain things I'm going to question even though I'm in the medical field. But we can't just sit here and say, oh, well, that happened such and such long ago. We have to acknowledge that it did happen such as the Tuskegee experiment, such as different experiments with people incarcerated that minority were African American even there was recently one that was drugs that was sent over to Kenya that caused people to be infertile. So we have to acknowledge it.” (3.2b, 4.2b)*
	4.2a.i Evidence-based findings	25	
	4.2b Transparency		
	4.2b.i Clear motive	9	
	4.2b.ii Open communication	21	
	4.2b.iii Safety and risk (security)	10	
	4.2c Acknowledgement of history	7	
	4.2d Pharmaceutical coverage	4	
Implementation	4.3a Advocacy	13	*“To do what it takes to accommodate the situation... .” (4.3a)* *“But you also have to meet that patient where they are because everybody is different...but it’s my job to advocate for you as well as help you to be able to achieve these things on your own.” (2.2a, 2.3a, 4.3a)* *“Yeah, I think that's important for sure. Maybe if it was someone that spoke their language, they may feel a lot more comfortable asking questions about the study. Maybe just someone who culturally comes from the same community that would be ideal. I don't know how often that happens though, but that would be amazing.” (2.3c, 4.3c.i)* *“Yeah. I think stakeholders need to feel engaged and I would say with research, you can't just go in and expect that they're going to want to participate. You need to establish that rapport with them first.” (4.3c.ii)* *“It's the very high [lead] and the minority populations, I will at least with the Parkland area, do not understand. So you need not only to have somebody that can interpret that to be recruiting them, but then also just the material itself, if you're just going to be handing it to them, have them be able to understand.” (4.3d.i)*
	4.3b Accessibility		
	4.3b.i Remote health	5	
	4.3b.ii Access to information	9	
	4.3c Community outreach		
	4.3c.i Hire diverse CHWs	13	
	4.3c.ii Build relationships with community (equal partnerships)	28	
	4.3c.iii Comprehendible educational material	14	
	4.3d Inclusion		
	4.3d.i Culturally translated material	6	
	4.3e Terminology revision	1	

### Facilitators influencing clinical research participation

Numerous key facilitators were identified as motivating factors across individual, interpersonal, community, and macro/policy levels by community (C) and stakeholder (S) groups. Monetary incentive (*n* = 31 and *n* = 28 for C and S groups, respectively) was constantly identified as the predominant motivator at an individual level (Table 2). However, the secondary facilitators differed at the individual level, where personal health knowledge (*n* = 26) was a motivator according to the community group and safety (*n* = 18), specifically for clinical studies involving drugs and devices, from the stakeholders’ perspective. Across both community and stakeholder groups, trusted relationships (*n* = 21 and *n* = 44 for C and S groups, respectively) emerged as the substantial motivator at the interpersonal level (Table 3). This encompassed trust in their primary care providers, community leaders, and the clinical research team. At the community level, health improvement was uniformly identified as the principal facilitator, with an emphasis on community health (*n* = 28 and *n* = 21 for C and S groups, respectively) (Table 4). However, the community and stakeholder groups expressed differing priorities at the macro/policy level (Table 5). Transparency, especially regarding safety and risk (*n* = 10), was identified as a key motivator for community members. In contrast, stakeholders highlighted knowledge from evidence-based findings (*n* = 25) as a notable facilitator, then transparency by open communication (*n* = 21).

### Implementation strategies for improving diversity in clinical research

Various implementation strategies by community members (C) and stakeholders (S) were identified across the four social levels of the SEM. Self-accountability (*n* = 15 and *n* = 5 for C and S groups, respectively) was perceived as the prioritizing implementation strategy at the individual level (Table 2). Community members and stakeholders both voiced that this human characteristic can be strengthened within individuals to help them become more competent with taking healthcare decisions as well as participate in clinical research. Identifying with the individual (*n* = 10 and *n* = 39 for C and S groups, respectively) was highlighted as the leading strategy at the interpersonal level (Table 3**)**. However, at the community level, proposed implementation strategies varied among community members and stakeholders. Community members identified awareness and education (*n* = 31 and *n* = 30, respectively) while stakeholders emphasized community outreach (*n* = 50) (Table 4). Yet, both community members and stakeholders converged on the importance of community outreach, specifically fostering or building long-term relationships with the community (*n* = 11 and *n* = 28 for C and S groups, respectively), as a pivotal implementation strategy at the macro/policy level (Table 5). Additionally, community members emphasized accessibility, particularly access to information (*n* = 12), as an important implementation strategy at the macro/policy level.

## Discussion

Low enrollment of diverse participant populations leads to disadvantageous gaps in scientific knowledge, especially in pharmaceutical trials, development of optimal clinical care practices, and areas of disease prevention and management. When looking to improve participation of UREG in clinical trials, it is crucial to account not only for the individual but economic, political, physical, technological, and socio-cultural influences as well [27]. Thus, to obtain a holistic perspective from diverse UREG, our study included primarily Black/African American and Hispanic participants, as well as those of or serving lower-income backgrounds and medically underserved communities. Using the SEM as framework [28, 29], we identified key barriers, facilitators, and implementation strategies stratified across the individual, interpersonal, community, and macroeconomic/policy levels to understand how clinical trial diversity could be improved.

### Barriers hindering clinical trial participation

We found that fear is a major contributor to community members’ hesitancy to participate in clinical trials at the individual level, which corroborates with other studies [30, 31]. Fear in clinical trial participation stems from the apprehension of becoming a “guinea pig” [15, 32], where individuals believe that they will become test subjects of a first-in-human study. The fear of “possible unknown side effects” and potential harms associated with treatments [30] is especially prevalent among those belonging to UREG, most often leading to outright declination of clinical trial participation [33]. Mistrust, which arises from a multiplicity of factors, including historical injustices, conspiracy beliefs, power imbalances, and lack of representation [34, 35], was also determined to be a key barrier. Like our community members, research stakeholders also recognized that fear and mistrust are significant impediments in clinical trial engagement at the individual level. This fear is often elicited when individuals encounter research-related topics, and its impact is even more pronounced in the context of clinical trials, significantly deterring individuals from engaging in research activities due to concerns about potential risks, unknown outcomes, and perceived exploitation [36].

Next, we found that beliefs, including myths, misconceptions, and cultural variables, are the most significant barriers to clinical trial enrollment at the interpersonal level. Previous studies report that individuals’ attitudes and beliefs influence their willingness to participate in clinical trials [37, 38], where one’s misconceptions driven by health illiteracy, ultimately impede their enrollment into clinical trials [39]. Misconceptions stem from various underlying factors, such as viewing clinical trials as dangerous to their health, believing that clinicians only conduct clinical trials for self-gain, and perceiving that clinical trials disproportionately benefit the White population [40–42]. People from UREG may also hold negative race-associated attitudes resulting from a history of neglected care and systemic racism [32, 38, 43, 44]. Through the community members FGs, we observed that people from different cultures hold differing views on clinical research, oftentimes posing as barriers to participation [37]. For example, another study that compared the attitudes of patients in the US, rural China, and urban China found that while 21.6–32.4% of American patients had no concerns about clinical research participation, only 1.6–2.9% urban and 1.4–10.7% rural Chinese patients shared the same attitude [45]. Two separate studies identifying participation barriers for Hispanic/Latino and South Asian communities found that barriers, such as fear of deportation and language dissimilarities, impacted both communities differently [46, 47].

Mistrust toward clinicians and other healthcare providers was an important barrier for clinical trial enrollment according to the community members. Healthcare professionals play a crucial role in facilitating or hindering representation in clinical trials, regardless of whether they identify with UREG themselves or not [48, 49]. Physicians’ conscious and unconscious biases toward UREG patients severely undermine their trust and impact their willingness to participate in clinical research [50]. As a result, having been the primary target of historical discrimination, Black individuals in particular possess higher mistrust in health care professionals [51], where they do not trust that their physicians would fully explain details of a clinical trial [33].

Stakeholders, like the community members, pinpointed cultural beliefs and misconceptions, including pervasive myths, as primary barriers at the interpersonal level. The term “guinea pig” was especially impactful, and such beliefs often perpetuated across generations, contributing to a stereotype that influences how UREG perceive research [52]. Additionally, cultural factors are pivotal at this level [10]. Our participants, particularly Hispanic individuals, revealed that cultural norms like the tendency to remain passive in unfamiliar situations negatively affect their engagement with clinical research. Stakeholders further identified the lack of awareness, characterized by insufficient understanding of various aspects of clinical trials, including their availability, objectives, procedures, and potential benefits and risks, as a critical barrier [15, 53].

Our current findings report that negative historical examples highly impact clinical trial participation at the community level, especially among UREG. Participants are often skeptical due to past ethical breaches and dehumanizing treatment within their communities. Black participants in our FGs referenced historic examples of human rights abuses in clinical research in the United States (US), such as the US Public Health Service (USPHS) Untreated Syphilis Study at Tuskegee [54]. Cases like Henrietta Lacks, thalidomide babies, and human experimentation with radiation during World War II continue to have harmful implications on individuals’ perspectives toward clinical trials [55–57]. The legacy of experimentation on UREG remains a barrier to trust medical research and participation in contemporary trials. Lack of knowledge was also a key barrier reported by community members. A study conducted in 2020 reported that 41.3% of US adults do not know about clinical trials [58], and those belonging to UREG are even less likely to be aware [53, 58–61]. Other individuals may be aware of ongoing research yet are unsure about their eligibility status [62, 63]. Surprisingly, studies report that underrepresented minorities are just as likely as their counterparts to consent if they were offered a trial [63–65], defying the common misconception that UREG are disinterested in research and will not partake in studies.

Stakeholders also acknowledged several barriers which impact clinical trial enrollment at the community level. Time, specifically the duration and commitment required for participation, may significantly influence an individual’s willingness to engage in clinical research. Stakeholders observed this as an obstacle in everyday clinical practice, and it is more pronounced among UREG [66]. Factors, such as economic constraints, employment obligations, and familial responsibilities, can exacerbate the impact of time-related barriers [15, 66] for those of underserved communities, making the demands of clinical trials feel more burdensome and less feasible. Additionally, stakeholders identified language, lack of knowledge and mistrust as secondary barriers. Difficulty in communication, mostly due to language differences, may prevent effective understanding of the research, leading to lower participation rates [67]. Insufficient knowledge about clinical trial procedures and benefits also leads to reduced enrollment [68].

At the macro/policy level, mistrust – encompassing distrust toward medical, pharmaceutical, and federal institutions – was the most frequent barrier reported by both community members and stakeholders. Distrust of the health care system in entirety is fueled by wasteful spending and 250,000 approximate deaths resulting from medical errors each year [10]. Additionally, medical mistrust is deeply rooted by systemic racism and is prevalent among UREG [69]. For example, a study in California reported that medical mistrust was 73% higher among Black adults and 49% higher for Hispanic adults as compared to White adults, where perceived discrimination was associated with 98% higher odds of medical mistrust [70]. Stakeholders reported that these issues contribute to a general skepticism among minorities who question the intentions and practices of the medical system and steer away from clinical trial enrollment.

Distrust in the pharmaceutical industry corroborates with a nationally representative study including 2,867 individuals at risk of cardiovascular disease, of which an alarming 60% adults do not trust pharmaceutical manufacturers [71]. Pharmaceutical mistrust is often the result of the interplay between multiple factors, including the perception of pharmaceutical companies as profit-seeking, promoting off-label marketing, and concealing crucial data [72]. It is believed that pharmaceutical mis- and dis-information campaigns disproportionately target vulnerable populations [71]. Additionally, general distrust in our government has been prevalent for decades. Pew Research Center revealed that only 20% of people claim they trust the government and 65% believe the government officials seek to serve their own personal interest [73]. Historically, a racial divide in public trust in the government has existed, with populations of color possessing less trust than their White peers [74, 75]. Thus, there may be a direct association between public mistrust in the government and individuals’ hesitancy to participate in clinical trials.

In contrast to the community perspective, consent and study language were identified as secondary barriers according to researchers. Stakeholders noted that the complexity of study documentation often exceeds the comprehension levels of UREG participants. Educational attainment in low-income UREG populations frequently aligns with lower reading levels [76]. Consequently, research documents unaccommodating these literacy levels or cultural contexts may discourage potential interested participants.

### Facilitators promoting clinical trial participation

We identified monetary incentive as the top facilitator at the individual level. Ethical controversies surrounding financial incentivization of medical research pertaining to inducements and coercion, particularly in low-income communities, may disproportionately lead to increased enrollment in clinical trials regardless of risk [77, 78]. Personal health knowledge, which encompasses a variety of factors, including understanding and being aware of health conditions, preventive health measures, health behavior knowledge, and access to healthcare resources, was recognized as another motivator by community members. Grunfeld et al. reported that participants sought a solution or cure for an ailment whilst others simply wanted better access to healthcare through cancer clinical trials [79]. The key facilitators identified by the stakeholder group – first monetary incentive, then safety, and last personal health knowledge – closely overlapped with those addressed by the community group (first monetary incentive, then personal health knowledge, and last safety) at the individual level. Leveraging basic facilitators, like monetary incentive and safety, is oftentimes overlooked by researchers, and therefore, it is crucial to involve community stakeholders and members throughout a clinical trial to understand the needs of young adults and UREG [80, 81].

At the interpersonal level, the importance of having trusted relationships was found to be a key facilitator among both community members and stakeholders. Trust is a foundational aspect of patient-provider relationships, which has the power to influence an individual’s decision to participate in research [82–84]. Having culturally competent and bias-aware healthcare providers is equally important in establishing a trusted connection. Additionally, trusted agencies, such as faith-based institutions [85], community health workers [86], and community organizations [87], further facilitate clinical trial enrollment and retention of UREG. These agents/agencies serve as effective liaisons between researchers and individuals on the foundation of trust, loyalty, relatedness, security, knowledge, and need [10].

Aligned with altruism – one’s desire to participate in research for the benefit of others [30, 79, 88] – improvement of community health and generational health were the top facilitators at the community level. These motivators function alongside personal health knowledge and family health knowledge to improve the wellbeing of a larger group. In our study, many community members felt compelled to participate simply because research outcomes may benefit their communities or future generations. Developing strategic community-focused interventions within key areas of need will likely result in increased participation leading to impactful outcomes and achieving the intended goals of public health research [89]. In addition, community engagement increases inclusion of UREG through transdisciplinary population health research [90]. Applying community-oriented models and frameworks ensures active engagement, promoting increased enrollment of underrepresented communities in clinical studies [91].

At the macro/policy level, transparency of safety and risk (security), maintaining open conversation, and clearly communicating motives were important according to community members. In our study, participants expressed the desire to be informed about the research outcomes of studies in which they participated, and transparency seeks to address the deep-rooted, multi-level mistrust expressed by individuals. Approximately 50% of clinical trial results are not published in peer-reviewed journals [92], which can been addressed by introducing new guidelines and policies to enhance transparency in the research process [92, 93].

Contrary to the perspectives of the community members, stakeholders pointed at knowledge through evidence-based findings as a significant facilitator at the macro/policy level. Majority of findings include those of European ancestry, and race and ethnicity have only recently been highlighted for genetic studies [83, 94]. This limitation must be overcome to better understand effects of investigational drugs and therapies for high-risk groups in clinical research [95–97]. Another motivator identified by stakeholders was transparency via open communication, then safety and risk (security) [98]. The NIH has taken measures to improve transparency across the research enterprise by developing a systematic process, one being clinical trial registration (ClinicalTrials.gov) and summary results which are open to public [99]. Additionally, the FDA published its first Drug Trial Snapshots beginning in 2015, which reports data sets of demographic characteristics of study participants, including trials from all approved drugs as well as NIH-funded clinical research and Phase III clinical trials [100]. Despite these approaches prioritized by federal agencies, systematic reporting of participation in clinical research and clinical trials remain inconsistent and limited [100].

### Implementation strategies proposed by community members and research stakeholders to improve clinical trial diversity

Self-accountability was identified as a key implementation strategy to improve clinical trial participation at the individual level. This sense of responsibility is inherently promoted through social interactions between healthcare providers and patients, and it is closely related to the concepts of competence (feeling of understanding), autonomy (having the jurisdiction to make informed decisions), and relatedness (feeling connected to others) [101]. Additionally, improving individual awareness of clinical trials is crucial. Effective, personalized dialogue between patients and providers facilitates individuals’ awareness of their role in clinical trials, thereby reinforcing their motivation to participate and contribute to research efforts. Although patients express interest in research, the majority have never had clinical trials discussed with them by their primary physician [102]. This gap in awareness may partly be attributable to inadequate communication, which includes both insufficient patient engagement and a lack of provider knowledge regarding available trials and eligibility criteria.

At the interpersonal level, a feeling of identifying with one’s healthcare provider or research team members through similarities in race, ethnicity, sex, age, and personal beliefs (i.e., religious views, cultural attitudes) is crucial [103]. Additionally, personalized communication through targeted outreach improves patient connection and willingness to participate in clinical research by addressing individual needs and enhancing perceived relevance. Clinical trial information should be tailored to align with an individual’s background, ensuring that it is both accessible and culturally relevant, thereby stimulating research engagement [104].

Multifaceted approaches are needed to mitigate the psychological harm stemming from historical mistreatment at the community level. Educating participants about how past wrongdoings have informed current ethical standards and regulations may rebuild lost trust [105]. Additionally, establishing collaborative partnerships with community organizations and incorporating cultural competency into research design through Community-Based Participatory Research strategies ensures that studies are both relevant and respectful of community values [91, 98, 106]. Equitable conversations throughout the research process via input of community members can identify the needs and struggles of study participants as well as strengthen trial designs and recruitment and retention approaches [107–109]. Furthermore, at a systemic level, integrating community feedback and perspectives into policy and practice ensures that interventions are relevant and responsive to the needs of diverse populations [110].

In this manuscript, we summarized findings from aggregated data and focused on generalizable barriers and facilitators for overall UREG participants. Our recent publication, which explored this focus group data, described differences and alignments across demographic groups and was thematically framed around mistrust [69]. A prominent difference was that the Black participants oftentimes cited the Tuskegee study whereas the Hispanic participants mentioned cultural norms, such as language barriers, identifying with who is recruiting, and challenges in research commitment while serving as independent caregivers for their family when discussing factors to participation. Both groups closely aligned when mentioning the fear of becoming the ‘guinea pig’ or ‘lab rat’ for research, where participants felt they did not know what they are getting involved with or what they would be given. Stakeholder and community member FGs were found to be more similar than different, suggesting that race and ethnicity may impact clinical trial participation more significantly than socioeconomic levels.

A significant limitation of this study is the substantial focus on only the perspectives of Black/African American and Hispanic communities of urban Dallas, restricting generalizability of findings to other UREG who may have different experiences with clinical trial participation. Additionally, the qualitative study was designed using the SEM framework, which may have voided other key components that impact patient diversity in clinical trials. Lastly, focus groups included mostly those of lower socioeconomic status, based on education level and household income, to best capture barriers and facilitators of medically underserved populations. Further exploration of perspectives of those from different socioeconomic levels may be worth investigating to better understand how perspectives of UREG populations may vary.

## Conclusion

Our study provides new insights into understanding the fundamental barriers, facilitators, and implementation strategies to increase clinical trial participation. Prominent findings emphasize rebuilding trust to bolster research integrity, building relationships through community engagement, increasing awareness about clinical research, and leveraging culturally tailored resources to promote inclusiveness. Overall, our work provides useful key components to improve representation in clinical trials, which holds promising potential to fill pivotal gaps in scientific knowledge. Ultimately, equitable clinical research participation is crucial for advancing health equity and informing public health policies, which will further ensure that translational research addresses the needs of all communities.

## Supplementary Information

Supplementary Material 1.

## Data Availability

All data generated or analyzed during this study are included in this published article.

## Abbreviations

C: Community member group
FG(s): Focus group(s)
NTCHC: North Texas Community Health Coalition
S: Stakeholder group
SD: Standard deviation
SEM: Socioecological model
UREG: Underrepresented racial and ethnic groups
