# Supplementary material for: Mapping the path to diversity in clinical trials: a qualitative study of community member and stakeholder perspectives
Source: BMC Public Health. 2025 Dec 9;25:4238. doi: 10.1186/s12889-025-25574-z (PMC12690815; doi:10.1186/s12889-025-25574-z)
Supplement: Supplementary file 1 — Supplementary Material 1. [file 12889_2025_25574_MOESM1_ESM.docx]

**Focus Group Guide for Community Members**

1. In your own words, please tell me what you think of when you hear the word ‘research’.
2. Which of the various types of research studies would you feel **most comfortable** participating in? Why?
3. *Prompt: What type of research studies would you* ***not feel comfortable*** *participating in? Why*? (i.e., *Clinical trial, Drug trial*)
4. How do you think Research can help your community, if at all?
5. *Prompt: How do you think your community feels about clinical research?  Pharmaceutical or drug research?*
6. What are some of the barriers to participating in research?
7. *Prompt*: *Do cultural and language issues influence your decision to participate in research*?
8. *Prompt: How about, time, transportation, etc***.?**
9. What would motivate you to participate in a clinical trial?
10. *Prompt: What types of incentives do you think would motivate people in your community to participate*?
11. *Prompt: What about pharmaceutical/drug trials*?
12. What do you think other people, like you, would need to help them choose to participate in a clinical trial? How about in a pharmaceutical/drug trial?
13. *Prompt: Safeguards/ confidentiality, if not discussed*
14. If you had to get the word out about the benefits of clinical trials to your community and helping recruit community members for clinical trials, how would you do it and what would you do?

**Focus Group Guide for Research Stakeholders**

1. In your own words, please tell me what you think of when you hear the word ‘research’.
2. Which of the various types of research studies would you think individuals in your community feel **most comfortable** participating in? Why?
3. *Prompt: What type of research studies would they* ***not feel comfortable*** *participating in? Why*? (i.e., *Clinical trial, Drug trial*)
4. How do you think research can help the community you serve, if at all?
   1. *Prompt: How do you think your community feels about clinical research?  How about pharmaceutical or drug research?*
5. What are some of the barriers (reasons) that keep individuals in the community you work with from participating in research?
6. *Prompt*: *Do cultural and language issues influence your decision to participate in research*?
7. *Prompt: How about mistrust?*
8. *Prompt: What about time, transportation, etc.?*
9. What would motivate the community you work with to participate in clinical trials?
10. *Prompt: What types of incentives do you think would motivate people in your community to participate*?
11. *Prompt: How about incentives for participating in pharmaceutical or drug trials*?
12. *Did we miss anything***?**
13. What do you think individuals in the community you serve would need to help them choose to participate in a clinical trial? How about in a pharmaceutical or drug trial?
14. *Prompt: Safeguards/ confidentiality, if not discussed*
15. If you had to get the word out about the benefits of clinical trials to your community and helping recruit community members for clinical trials, how would you do it and what would you do?

**Survey**

1. What is your Age? (In Years) ___ ___
2. What was your assigned biological sex at birth?

| Female | None of these describe me |
| --- | --- |
| Male | Prefer not to answer |

1. Are you of Hispanic, Latino, or Spanish origin?

| No, not of **Hispanic, Latino, or Spanish origin** | Yes, **Puerto Rican** |
| --- | --- |
| Yes, **Mexican, Mexican Am., Chicano** | Yes, **another Hispanic, Latino, or Spanish origin** – Print, for example, Salvadoran, Dominican, Colombian, Guatemalan, Spaniard, Ecuadorian, etc. __________________ |
| Yes, **Cuban** |  |

1. What is your race? Mark one or more boxes.

| **White** – Print, for example, German, Irish, English, Italian, Lebanese, Egyptian, etc. ______________ |
| --- |
| **Black or African Am.** – Print, for example, African American, Jamaican, Haitian, Nigerian, Ethiopian, Somali, etc. ______________ |
| **American Indian or Alaska Native** – Print name of enrolled or principal tribe(s), for example, Navajo Nation, Blackfeet Tribe, Mayan, Aztec, Native Village of Barrow Inupiat Traditional Government, Nome Eskimo Community, etc. _____________ |
| Chinese |
| Filipino |
| Asian Indian |
| Vietnamese |
| Korean |
| Japanese |
| **Other Asian** – Print, for example, Pakistani, Cambodian, Hmong, etc. ____________ |
| Native Hawaiian |
| Samoan |
| Chamorro |
| **Other Pacific Islander** – Print, for example, Tongan, Fijian, Marshallese, etc. _____________ |
| **Some other race not listed above** – Print race of origin _____________________ |

1. What is the highest grade or level of school you have completed or the highest degree you have received?

| Never Attended/Kindergarten Only | 1st Grade | 2nd Grade |
| --- | --- | --- |
| 3rd Grade | 4th Grade | 5th Grade |
| 6th Grade | 7th Grade | 8th Grade |
| 9^th^ Grade | 10th Grade | 11th Grade |
| 12th Grade, No Diploma | High School Graduate | GED Or Equivalent |
| Some College, No Degree | Associate Degree: Occupational, Technical, Or Vocational Program | Associate Degree: Academic Program |
| Bachelor's Degree (Example: BA, AB, BS, BBA) | Master's Degree (Example: MA, MS, MEng, MEd, MBA) | Professional School Degree (Example: MD, DDS, DVM, JD) |
| Doctoral Degree (Example: PhD, EdD) | Prefer not to answer | Don't Know |

1. In total, how many people live in your household, including you?

| 1 | 2 | 3 | 4 | 5 | 6 |
| --- | --- | --- | --- | --- | --- |
| 7 | 8 | 9 | 10 | More than 10 |  |

1. What is your best estimate of the total income of all family members from all sources, before taxes, in the last year?

| $0 to less than $20,000 | $65,000 to less than $80,000 |
| --- | --- |
| $20,000 to less than $35,000 | $80,000 to less than $95,000 |
| $35,000 to less than $50,000 | $95,000 or more |
| $50,000 to less than $65,000 |  |
